# Primary care services in the English NHS: are they a thorn in the side of integrated care systems? A qualitative analysis

**DOI:** 10.1186/s12875-023-02124-3

**Published:** 2023-08-30

**Authors:** Claire Mitchell, James Higgerson, Abigail Tazzyman, Will Whittaker

**Affiliations:** 1 School of Health Sciences, Faculty of Biology, Medicine and Health, University of Manchester, Oxford Road, Manchester, UK; 2 Faculty of Biology, Medicine and Health, University of Manchester, Oxford Road, Manchester, UK; 3 University of Sheffield, Sheffield, UK

**Keywords:** Integrated care systems, Primary care services, Integration, Health care, Social care, Qualitative research

## Abstract

**Background:**

As integrated care systems are embedded across England there are regions where the integration process has been evaluated and continues to evolve. Evaluation of these integrated systems contributes to our understanding of the challenges and facilitators to this ongoing process. This can support integrated care systems nationwide as they continue to develop. We describe how two integrated care partnerships in different localities, at differing stages of integration with contrasting approaches experienced challenges specifically when integrating with primary care services. The aim of this analysis was to focus on primary care services and how their existing structures impacted on the development of integrated care systems.

**Methods:**

We carried out an exploratory approach to re-analysing our previously conducted 51 interviews as part of our prior evaluations of integrated health and care services which included primary care services. The interview data were thematically analysed, focussing on the role and engagement of primary care services with the integrated care systems in these two localities.

**Results:**

Four key themes from the data are discussed: (i) Workforce engagement (engagement with integration), (ii) Organisational communication (information sharing), (iii) Financial issues, (iv) Managerial information systems (data sharing, IT systems and quality improvement data). We report on the challenges of ensuring the workforce feel engaged and informed. Communication is a factor in workforce relationships and trust which impacts on the success of integrated working. Financial issues highlight the conflict between budget decisions made by the integrated care systems when primary care services are set up as individual businesses. The incompatibility of information technology systems hinders integration of care systems with primary care.

**Conclusions:**

Integrated care systems are national policy. Their alignment with primary care services, long considered to be the cornerstone of the NHS, is more crucial than ever. The two localities we evaluated as integration developed both described different challenges and facilitators between primary care and integrated care systems. Differences between the two localities allow us to explore where progress has been made and why.

## Background

Integrated Care Systems (ICSs) within the NHS in England involve primary, secondary and social care services and other partners working together to provide health and social care to the local population.

In July 2022, following the passing of the Health and Care Act 2022 [1], 42 ICSs were created to cover the NHS region in England on a statutory basis. This can be seen as the culmination of a relatively rapid shift in recent years towards ICSs in delivering health and social care services to populations with complex health needs.

ICSs, as per the Health and Care Act 2022, comprise an integrated care board with oversight of planning and resource allocation, and an integrated care partnership focused on local population health priorities and service delivery [2]. Although the shift to ICSs is a national policy, the expectation is for places and neighbourhoods to deliver their ICS according to local priorities and needs, with a heterogeneous set of systems anticipated [3, 4]. ICSs are a fundamental change to previous organisational healthcare structures where there has been a division between organisations as well as separation of commissioners and providers [5, 6].

ICSs are being developed against a backdrop of complex challenges in organisational structure and commissioning which is particularly evident in existing Primary Care Services (PCSs) [7, 8]. There is a complex and well-documented history of commissioning PCS which could affect the success of the integration agenda [9]. The emphasis on integrated care has led to guidance for local organisations to work together across geographical localities to produce a place-based partnership that considers the needs of the local population and works towards an agreed shared vision of collaboration and integration [10, 11]. A key part of the ICS architecture is the development of Primary Care Networks (PCNs). These have commissioning oversight of some primary care services (PCSs) such as general practice services ensuring the functions of primary care support integration [12]. Primary care is therefore a key player in planning and resource allocation, as well as service delivery and population health protection.

PCNs are where primary care services comes together to operate at scale through place-based (neighbourhood) networks of primary care practices working together in federations or merging [13]. The intention is that staff will work across practices and in an integrated way with other services. The Kings Fund report states that primary care is the key to development of the ICSs and that the development of primary care working at scale can support this engagement [7].

This paper brings together findings from two separate research projects around the formation of voluntary integrated care partnerships (ICPs) in two localities in England, as part of the wider ICS for that region. We have re-analysed the data to examine the specific challenges related to barriers and facilitators of integrating primary care services within these ICPs.

These evaluations around the formations of two ICPs (localities A and B) in separate locations of England, observed different approaches to voluntary integration and introduced integrated elements at varied time points in response to existing services and local assets. Both of these localities took an approach where reorganisation across the whole system resulted in the setting up of co-located neighbourhood teams, which we term place-based integration.

Locality A: Introduced a single commissioning system, a single local care organisation for community health and social care services and a single secondary care service so three inter-dependent parts. This locality was in the early stages of co-locating neighbourhood teams. Interviews conducted April to November 2018, at the time of the original evaluation.

Locality B: Introduced a single-commissioning function as part of the place-based working and introduced specific initiatives from transformation funding. Transformation funding schemes involved professionals from across co-located teams, primary and secondary care. Transformation schemes are where public funding has already been used to create standalone, integrated, solutions to a particular problem for that locality. Established co-located neighbourhood teams were already in place. Interviews conducted 2019–2020 (including during the Covid-19 pandemic), when the original evaluation was being carried out. Evaluation of both these localities made evident the challenges to the integration of the PCSs.

### Research context

In this research we re-visit our evaluations of integration in these two locations and our objective is to identify the main themes emerging from the interviews around the barriers and facilitators to the participation of PCS into the wider ICS. Through this we aim to highlight the key differences and commonalities experienced in relation to primary care and ICS formation in two localities with different approaches to the process. Specifically the research question to explore how PCSs within the development of two ICPs might impact the development of the ICSs.

### Contribution to the field

This paper provides a contribution to the under-researched area of the role of PCSs within the ICS. The two differently designed services, PCSs and ICPs, are now expected to develop and work together seamlessly within an ICS but this is not always straightforward. We describe some of the challenges relevant to the development of ICSs and what factors may foster the relationship between PCSs within the ICSs as this work continues to develop as part of the NHS long term plan [14] and Health and Care Act [1].

## Methods

We carried out semi-structured interviews with staff involved in the two localities, from both operational and strategic levels. Staff interviewed were from health and/or social care backgrounds and from both secondary care and PCSs. The interviews were conducted with a schedule of broad questions designed to gain an understanding of the context, barriers and enablers to integration from the interviewee’s perspective. The sampling strategy included both purposive and snowball sampling, in both localities, to ensure an even number of people in health and social care which also included interviewees from PCSs. In locality A, 24 interviews (1-24 A) were carried out between April to November 2018 at the time of the original evaluation. In locality B, 27 interviews (1-27B) were carried out between 2019 and 2020 (including during the Covid-19 pandemic). In total 51 interviews were carried out (19 from social care, 33 from healthcare and of these 13 were from PCSs and 20 from secondary care services. Professions not identified to preserve anonymity).

All semi-structured interviews were carried out in person by either one or two interviewers (conducted by authors 1, 2 & 3) either at the individuals’ place of work and during the Covid-19 pandemic, over the phone at any location suitable for the interviewee. The interviews lasted between 45 min and 1 h 30 min. The interviews were all audio-recorded, with participants’ written consent, transcribed verbatim and anonymised before being transferred to NVivo11 software to store and manage the data [15]. Field notes were made during and after interviews.

Analysis was approached thematically. A coding framework was developed by the authors through interpretation of the data and a previous scoping review of the literature, we focused on challenges and facilitators relating specifically to PCSs or PCNs [16]. Further codes were added to the framework inductively as appropriate and then coded across all transcripts [17, 18] by all the coders (authors 1, 2 &3) during discussion meetings. Once all the transcripts were coded the findings relating to each locality were in addition compared to identify similarities and differences across them. Four key themes from the data are discussed: (i) Workforce engagement (engagement with integration), (ii) Organisational communication (information sharing), (iii) Financial issues, (iv) Managerial information systems (data sharing, IT systems and quality improvement data).

Ethics approval from the University of Manchester Research Ethics Committee was granted for this research (Ethics MBS 2017-2979-4620 & PR UREC 2019-6082-12026) and Health Research Authority approval (IRAS project ID 238,256 & 260,908).

## Results

In this paper we aim to provide an understanding of the barriers and facilitators to the involvement of the PCSs in two localities as they became ICPs. We report on some of the tensions that emerged between primary care and other aspects of the system as well as the factors that facilitated engagement.

### Workforce engagement

This theme covers data relating to the attitude of workforce members towards integration, relationships between those involved with work connected to integration, and engagement in the integration process.

Participants in both localities reported that strong working relationships, across health and social care and between primary and secondary care were crucial for the success of integration. Participants referred to both pre-existing and newly formed relationships in regard to this. A shared belief in the value of integration was key to creating a willingness for these relationships to develop and be maintained. As a result of such relationships across workforce connected to integration it was reported that existing practical and cultural boundaries between health and social care, as well as those between primary and secondary care were able to be overcome to some degree. In contrast, where relationships were weak or historically difficult, at both individual and professional level, it was seen to contribute to a lack of willingness to buy into integration:

*“yes, the money might have gone into the hospital but the hospital spent it on community services, which people in neighbourhoods benefitted from. So therefore those patients are the same patients that GPs [General Practitioners] look after.” (4B)*

Historic poor relationships and barriers between secondary and primary care were repeatedly raised as barriers to integration and seamless working. One solution raised by many was bringing health and social care personnel together and explicitly discussing the different expectations and pressures in each which was considered to have led to improved relationships and understanding:

*“…there was practically no dialogue between primary care clinicians and secondary care clinicians. Practically none at all. Quite a lot of mistrust, built up over years….We had loads of GPs and hospital consultants who came into that programme on a Wednesday evening for eight weeks, where they learned about how to manage themselves through change. And in that, they met other people who were there and you start to break down those barriers. And fundamentally, you know, when people are just chucking rocks at each other over a wall, you get nowhere. When you’ve introduced people and everyone starts to understand a little bit more about other people’s pressures, then you get the ideas and we all get on”. (4B)*

Building relationships and breaking down barriers was seen in one of the locations as an opportunity to get to know other professional groups. There was a strong suggestion that sharing knowledge, understanding each other’s roles as well as the opportunity to work together more could improve relationships. Particularly the importance of shared learning in the workplace, joint and multi-disciplinary working, rather than the more passive hearing talks from other members of staff:

*“So I think it’s going to be more about understanding each other’s role in order to make integration work, if we professionals we take part in training education, that we know each other’s role, I think that would make things better, the only thing is making integration difficult or challenging is lack of understanding of each other’s role.” (19A)*

There was a sense that some professionals working in PCSs were unlikely to change their attitudes, and that it was known who would be more or less likely to resist change. There were some suggestions that with this existing knowledge, greater attention should be given to involvement and engagement in integration to improve workforce attitude and understanding:

*“…yet they [PCS professional groups] just didn’t see...I don’t know, it was just really sad, I don’t why they didn’t see the vision in the same way we saw the vision. I don’t know and there must be some fault in what I did that didn’t float their boat, but it certainly didn’t.” (1B)*

It was suggested by some that there was little interest or engagement with integration from the PCSs, a sense that people weren’t listening or not understanding the impact of the work on the health and wellbeing of the local population. This may well reflect how and when different schemes were introduced and explained:

*“So I know I’ve stood at a target meeting, which is like their education meeting for GPs, and I’ve done a presentation about a scheme or whatever and then a month later they deny all knowledge of knowing that that scheme ever existed.” (14B)*

The importance of relationships and a shared vision is highlighted by many of those involved in leading change and developing integration with a sense of having to build these foundations before integration can work. There is a sense that the previously discussed workforce attitudes then affect the success of whether people can work together and share the same vision:

*“So, an awful lot of the work that we’ve done, has been about relationships, and it’s been about hearts and minds, and it’s been about having a team of people who are very values based, and very collaborative in their style of working.” (19A)*

Some suggested that PCSs do not always engage fully with integration which tends to put the responsibility of this on those working in ICPs. On the other hand there is a strong sense that the PCSs were not always involved in the early decision making but expected to deal with whatever is decided about them going forward whether this involves staffing resources or funding. Building relations with primary care was certainly seen by many of our interviewees as a priority:

*“Again, the GP community is actually first port of call with a patient, we’re in this together, with our GP members, saying, look, this is what we’re doing for the hospital, we’ve got to try. Now they needed a lot of convincing, it wasn’t easy, and these are some of the choppy waters, we had to bring members on board, we had to bring the political influence on board.” (9A)*

Trust was raised as an important aspect, where a lack of trust can hinder relationships, which in turn hinders integrated working. Trust referred to the responsibility of patient care as well as trusting other professionals to do what they say they will do within the care pathway:

*“And I think the trust issue is something we’ve got to work on with our GPs because that’s the way to build the relationship – if they don’t trust us we’ll never build the collaborative relationship.” (14A)*

Trust was seen as key to integrating PCSs with wider health and social care services. There was an understanding that trust is particularly important when PCSs really have a different relationship with, and responsibility to the people they work with compared to those in secondary care:

*“Some of the GPs, you know, some of them were quite hard to deal with initially, and I get it, you know. They’re their patients, they have the ultimate care, and they still have the ultimate care." (18A)*

### Organisational communication

Another clear theme related to how ICPs are developed and set up and how effective communication is before, during and after the formation of the organisation. Transparency around this process and true engagement of all those involved was considered necessary to build relationships, trust and belief in the vision. Channels of communication are a key part of successful integration and that these are made clear to the whole of the workforce:

*“I think relationship are much closer than they used to be. We still have differences of opinion but, you know, that’s the same all over the country between primary and secondary care... I think communication routes are much better than they used to be. I think everybody is broadly signed up to where we’re all going, in terms of our direction, and understand that, you know, nobody is an island and we’ve all got to work together. Yes, so I think it’s been very positive.” (4B)*

Barriers and poor relationships between primary and secondary care can restrict integration, whereas clear channels of communication can improve people working together within ICSs:

*“So in the old system, once a patient had gone to hospital, the GPs would be very much of the opinion, well, that patient is now in the hospital and it’s not really our responsibility. And now we have a situation where if a patient’s in hospital, there will be communications between the hospital and the GP practice whilst they’re in hospital, saying, look, this patient’s been in hospital for 30 days, are you aware of any social barriers? Are you aware of anything we can do to try and get the patient out?" (4B)*

Where people highlighted conflict within the organisation of integrated services, this was often caused by situations being imposed on departments or services without consideration of current local or clinical workforce set ups. There are also some suggestions that ICPs cannot necessarily be replicated across the locality, or geographical region, any more than they can be replicated across the country. There are always local needs and differences as well as assets in different locations and this could be incorporated at an early stage of development if all stakeholders are included:

“…*it feels like it’s so hospital-centric, the whole system, you’re either in hospital or out of hospital services. People have short episodes of their lives hopefully in hospitals, then they live in their own homes.*“ *(2 A)*.

One of the challenges to engagement with the PCSs is the difficulty in getting clinicians involved in that early decision making without this having an impact on clinical care. This also relates to the costs associated with running a PCN and whether development type work can be incorporated into this, when resources are limited and services have clinical targets to meet:

*“So, they’d [the GPs] got scheduled patients at nine o’clock and if I’d pulled them all into a meeting at nine o’clock every month on a Friday, then I’d lose a lot of activity in GP land which the GPs wouldn’t like. So, how do I or we as a wider health economy make sure those people are engaged with other stuff which is going on?” (15B)*

PCSs, despite the difficulties in engagement and co-design need to feel involved in decision making and developing services that they feel will benefit the practice and the local population. We found that the introduction of the PCNs did affect some of the integrated schemes that were already up and running in one of the locations we examined. The contracted services changed and PCNs were expected to pay for services they had not been involved in setting up and were not necessarily sure they needed. This had huge implications for professional groups where people were made redundant as seen as ‘not cost effective’ by the PCNs who then inherited an expensive service that they were not prepared to pay for. This led to bad feeling between professional groups and the PCNs:

*“I think it needs to be looked at, where does the GP fit within this integrated working? What is their commitment to joining with integrated working instead of keeping separately on their own and we’re forever banging on their door and saying will you join in with us?” (12A)*

PCS involvement needed to be financially sustainable as this was considered a crucial part of integration. PCS staff being involved in integrated services and actively engaging with this would be seen as essential to deliver seamless integrated care. GP views from our interviews suggested the benefit to involvement with integration out-weighed the time taken. It was felt by other professions that GPs may view involvement in integration as leading to an increase in responsibilities and taking time out of an already unmanageable workload:

“*…for us [community nurses] working in the community the GP is at the heart of everything. And if a GP is not part of your integrated team, what do you call integrated? I think it needs to be looked at, where does the GP fit within this integrated working?” (19A)*.

There is a wider understanding that PCSs were under enormous strain and pressure to deliver services, while keeping finances under strict control. This is in addition to the potential to be expected to deliver more services. Working with PCSs at the earliest stage could avoid some of this conflict around imposing services without evidence to show benefit and potential time-saving:

*“Well, the GPs initially were like well, you’re [secondary care services] adding to my workload by saying this, and it took us a little while to demonstrate that actually we were avoiding a whole load [of work].” (25B)*

### Financial

Finance featured frequently as a topic in the interviews conducted. In some instances, participants stated a belief that the PCSs were too focussed on the finances, however there was an acknowledgement by others that the structure and set-up of PCSs meant they comparatively had to be focussed on the finances. This relates back in some ways to the theme around workforce attitudes, relationships and trust. Some presented a view that the PCSs were only seen as wanting to protect their finances with limited acknowledgement of financial pressures, while trying to deliver quality services:

***“****Yeah, there was no appetite to integrate ... In fact it was the opposite, they didn’t want us [secondary care services] in there at all and having attended some of these meetings that they had every month, it was obvious that there was an inherent hatred of the trust and they were seen as wanting to get money off GP services.” (1B)*

The conflict between secondary and primary care services came through in several contexts, particularly where one locality separated out these services and one locality tried to streamline the two. The sense of there being a financial disparity between what finances were given top priority were made clear by many of the interviewees. Some participants felt it was not so clear cut and that finances given to one area only could have wider ranging impacts on services:

*“There’s a lack of understanding around actually what a difference an investment in community services could make” (3A)*

There was also an understanding that the PCSs have to balance the books, pay staff and spend their budgets according to local need. This is how they were set up and organised so there was an understanding from many interviewees where they acknowledged PCSs did have to do this:

*“I think there’s mistrust for some reason even though not all GPs...I know some GPs are very money minded and what have you and partly we have to be because we’re [GPs] all running small businesses, aren’t we? And if we don’t think about our income then we can’t pay ourselves at the end of the year.” (1B)*

Integration and the various transformation schemes we looked at in our evaluation did often cost money and there was not always an easy way to show these payments had a cost benefit. In some situations they may not have saved money but may have improved care. When PCSs and ICPs have strict budgets and are measured on different metrics, this can be challenging to integration:

*“And also that other thing is, is that we’re constantly having to prove that we’re going to save money, so there’s all this thing about, what’s the cost benefit analysis? Not, actually, are we going to make a difference to people’s lives, are we going to improve the outcomes for individuals, and actually, they have a better experience of their contact with health and social care, but actually for every pound invested, how much did we save?“ (1A)*

As well as the expected financial outlay from the PCSs these were schemes that were being done to them rather than in partnership with them, with the potential for revenue loss. It was felt that the bidding process for some integration funds excluded GPs, and that engagement with GPs through integration could be tokenistic.

The other side of this is the view expressed in some interviews that, as business owners, GPs tended to prioritise money and the lack of financial remuneration for scheme participation might have been a barrier. It appears the benefits to primary care of the transformation schemes was not initially recognised within that sector, whether that was because it was poorly communicated or poorly received:

*“…with the intention of keeping them [patients] out of hospital and caring for them better in their own home. However, that didn’t meet their agenda because at the end of the day these GPs and GP surgeries are businesses and what they want is to make money.” (8B)*

### Managerial information systems

There was scepticism about the benefits of integration being felt in primary care or whether the benefits were really to improve secondary care data/stats. The standard metrics for success or improvement from integration are still acute care focussed which increases the feeling of secondary care being prioritised. The perception expressed was that these schemes were mainly intended to benefit acute care targets and make the hospital more sustainable:

“*…aims are reducing A&E admissions and stays in hospital and stuff. But the bigger thing is, we will talk about improving health outcomes, enabling people to live longer. Well, in terms of the prevention work, and some of the stuff that we’re doing, there’ll be no deliverables…we’re actually trying to rebuild relationships, or start new relationships, to make some of this stuff happen” (1A)*

Some GPs have taken hospital-based roles as part of the integration agenda in their locality, such as within certain integration schemes where GPs are based in emergency services for example. This provides primary care context to the discharge to assess model with the potential to reduce primary care pressures, but if this change is not communicated as such, it may appear more like these services taking over primary care resources. This was evidenced with some issues of redeployment of clinical staff to the hospital when required by schemes. This can also work the other way with services struggling to get embedded in primary care services as part of new integrated services:

*“It was very difficult because when we [secondary care services] started the service, we had some tremendous barriers, mainly because we were employed as an integrated care organisation and the GP surgeries would not allow us access to that system. Even getting a foot in the door at the GP surgeries to review patients was very, very difficult. It took us a long time to embed our service in these GP surgeries.” (8B)*

This viewpoint might also be validated by the metrics used to assess the impact of various transformation schemes within the locality, whereby many services were measured against bed days saved and changes in hospital activity:

*“There has been wider efficiencies across the system, and we don’t capture them all yet either. We do struggle to capture primary care data. So we can’t really quantify the impact some of these services have had on primary care. Some we can, but a lot of them we can’t really demonstrate how they’ve impacted primary care, even though we can confidently assume that they’ve impacted them but they just don’t collect data in the same way as the acute trusts do so we can’t get that.” (25B)*

Primary care focused metrics did not feature in many of the outcome measures for transformation schemes, and data were not provided in those circumstances where it was sought:

“*For us it’s how we collect the data that demonstrates, yes we work with people that we can enable self-care, but the complex people who have many complexities for many different reasons, if we don’t keep that intervention going they will end up in hospital. So how do we collect data about that and how do we demonstrate how it’s keeping people out of hospital?” (19A)*

It is not surprising that data sharing is a clear problem across all of our evaluations on integration. It continues to be problematic for PCSs and how they work with ICPs:

*“But, with the new GDPR it’s, kind of, now making things a bit more challenging….Because, when you’re requesting information and consent has to be given, so we have to do it in a way were maybe at that moment in time I’m not in contact with the person, therefore I’ll have to liaise with the doctors or the health professional that is working with that person…” (14A)*

This was one of the most commonly reported issues across the localities and continues to be a source of frustration and time wasting. There is a sense that higher level strategic leaders and managerial staff could go some way to addressing these issues by getting agreement at board level for staff to share the same data:

*“Two and a half years down the road now today I’m still waiting for data sharing agreements to be shared by the GPs in all the practices, so that I can have a productive working model. It is absolutely unbearable and causes a great deal of frustration. There was no working party created before we started in post to lay the foundations for us, so there’s immediately massive barriers that prevented us from developing a very good service.” (8B)*

In one example reported in our research about a particular aspect of a new scheme that was part of an ICP the leaders involved established a data sharing agreement at the earliest point. This was found to greatly improve the success of the scheme as all staff had access to the same data systems:

*“That if this was going to work and for us to be safe, make sure that patient was safe, we needed this information. And that was all set up from the word go, before we even tried it.” (18B)*

## Discussion

Throughout our two separate evaluations of how integration had been developed, in different ways in two localities, we found common challenges with how ICPs worked together with these pre-existing PCSs. There were clear complexities around how the individuals involved were working towards integration and how this was hindered where change was not collaborative or elicited involvement of all parties at an early stage. There were clear areas of conflict around the finances, particularly where the existing model of PCSs were at odds with the financial expectations of the ICPs. Unsurprisingly communication and data sharing between the localities and PCSs were challenging, as we found through all our evaluations between different health and social care systems.

Integration is considered to be the future of health and social care in England and embedded in the NHS Long Term Plan [14] and Health and care bill [2]. This does not necessarily mean it is straightforward or easy to accomplish [19]. In fact, a lot of the evidence highlights the complexities and challenges around integration and how different localities experience individual challenges according to the needs of the local populations [20, 21]. There is a wealth of evidence now around integrated care, but areas across England have not always incorporated existing learning or even evaluations of their own activity to improve the process [20]. This means replicating integration across areas does not work, although there have been examples where elements of good practice or specific schemes have been successful in other areas [22]. One of the key facilitators to integration in all locations seems to be a common belief or shared vision about the purpose and benefit [20, 23, 24]. There seems to be an understanding that having this same goal can support individuals to work together and build trust and relationships across professional boundaries and across the boundaries between health and social care as well as primary and secondary care [25, 26]. It became apparent in both of our evaluations that there can be conflict between previously established functioning PCSs, with an expectation that they should embrace integration and understand all the different elements to it. In some cases those working in PCSs did not feel they had been fully consulted early on or truly engaged in co-development. There was sometimes a sense that those working in primary care were obstructive or dis-interested but no real consideration of why this may be. Related research also suggests that knowledge or the strategy to integrate influenced the actions of the professionals involved [21]. Our findings did not highlight leadership as an issues unlike other research in this area [27], as well as our previous analysis and publication of the wider evaluation we carried out [28]. This may reflect the questions that were asked or the sense that we were exploring the day-to-day functioning of the teams, not particularly focussing on the leadership.

Communication across organisations is challenging in many ways and is apparent throughout many integration evaluations [20, 29, 30]. This seems to be the case from high level managerial to operational staff, across health and social care boundaries as well as between primary and secondary care and professional groups [28]. Poor communication can foster mistrust, it can affect the individual attitudes and relationships already discussed and all this can lead to greater conflict over financial resources. Communication relates to having access to other professional groups, being able to negotiate and deal with conflict and supports building relationships. It seems that some key aspects that support communication can support integration and understanding including a shared vision [20] and how leadership provides the environment to achieve this [27]. This includes easy access to other professionals and services across service boundaries such as primary to secondary care. This may be forums where cross boundary health care professionals get the opportunity to co-develop services and work together, as well as the physical co-location of teams from across component organisations of the ICS. This may also relate to having an understanding of each other’s professional and financial obligations through shared learning and shared work experiences where possible which has also been identified elsewhere [28]. Some of the legacies of previously encouraged competition between services, that can impact on successful communication and relationships, has been discussed in other research that supports our findings [31].

Finances can always be challenging particularly due to the historical complexities of funding between different NHS services and there is an expectation that ICSs will protect the partnership model of PCSs and the PCNs as independent contractor status [10]. This status was set up prior to the ICSs and in some ways there is inherent conflict that arises from financial expectations. Where integration has been perceived as ‘imposed’ on PCSs, or where integration has a financial outlay or potential financial burden in PCS this can be problematic on both sides [32]. Ensuring schemes are both value for money but also are aimed at patient care being the priority mean that both ICPs and PCSs will have differing needs and priorities. Financial implications are well documented and the limited resources in other integrated areas such as mental health support our findings [33]. Some of the services added an unexpected financial burden to the PCSs with no discernible benefit to the local population or sometimes this was a time burden which the PCSs were reluctant to deliver on. There was a clear belief from the PCSs that the integrated services had to offer value for money and the most important aspect is that they improved health outcomes, so improved longevity but also the quality of that longer life. Some of the schemes could show improved health outcomes to the local population with demonstratable benefit to PCSs, which was the best way to get PCSs support, both financial and time resources although demonstrating health improvement is challenging [34].

Integration, as shown in the majority of evaluations, is hindered by the challenges of different IT and data sharing agreements as is well documented in other research [20, 29, 30, 35]. This again goes across all boundaries, between health and social care, primary and secondary care. It is no surprise that these systems hinder the ability of professional groups to communicate, as discussed previously this can lead to relationships breaking down, a lack of trust and a misunderstanding of different priorities. Where integration has been successful, high-level agreements have been implemented early and sorted out prior to people needing to access the same systems and data. There are opportunities for national policy to facilitate the sharing of data between different sectors of what is intended, through ICSs, to be one cohesive system as described elsewhere [35].

The NHS continues to be held in high regard by the population of England and the development of the ICSs has to achieve health and social care that meets the needs of the population and responds to changing needs of each locality [36]. Integration has been talked about for many years and has been a challenge for many governments as they strive to further this goal. Now integration has to happen, it is to be embedded and mandated across the country [2, 14]. However there continue to be challenges, some areas are further ahead than others and some will achieve integration more successfully than others [31]. ICSs will continue to develop and evolve, to improve and find better ways of working together more effectively over time to improve the care and outcomes for the public. There is a growing body of evidence related to integration and this work offers a view of the key issues that are apparent between ICPs and PCSs.

Conclusions: ICSs will continue to develop despite the areas of challenge and potential conflict that we have found in our evaluations. It seems that ensuring both PCSs and ICPs continue to develop and improve services with shared involvement and an understanding of each other’s perspectives can support successful ICSs. This can lead to building better relationships, trust and breaking down barriers between professionals and services. This shared understanding will help explain the financial obligations inherent in both ICPs and PCSs which will allow greater transparency of discussion. Establishing easily accessible means of communication, shared IT and data can also support relationships and develop shared goals of integration to support the health and wellbeing of the people in that location. For those involved in developing ICSs across England, understanding where conflict arises and why can support their approach to this process.

### Strengths and limitations to this study

This study was carried out following our original evaluations of two ICP localities. It became apparent that there were specific issues related to PCSs and the impact this could have on ICSs that would benefit from further exploration. This means our original recruitment strategy was to evaluate the two ICP localities not specifically to examine PCSs and their role in ICS. There is a time lag between interviews due to the separate evaluations, and this may be a factor in the results we had. However, both ICPs were at different stages of development which could also add to the richness of the data we explored. One of the evaluations took place during the Covid-19 pandemic and many of the interviews were conducted over the phone in locality B. This may have affected the quality of the data in that locality compared to face-to-face interviews, as phone interviews do not always explore issues in as much detail.

## Data Availability

The data cannot be shared publicly due to stipulations in the ethics agreement but can be made available from the corresponding author upon reasonable request.

## Abbreviations

ICS: Integrated Care Systems
PCS: Primary Care Services
PCN: Primary Care Networks
ICP: Integrated Care Partnerships
GP: General Practitioner
